# Academia's class problem

**DOI:** 10.3389/frma.2025.1566023

**Published:** 2025-04-04

**Authors:** Thomas J. Spiegel

**Affiliations:** Faculty of Human Sciences, Waseda University, Tokyo, Japan

**Keywords:** class, classism, Marxism, university, academia, injustice, justice, research

## Abstract

The university has a problem with social class. Actually, it has two problems with class. The first one is that the university as an institution is still largely impervious to people from lower socio-economic backgrounds. In particular, people with middle and upper-middle-class backgrounds are overrepresented especially in very desirable tenured positions. This article offers a brief assessment of the problem, argues that more class diversity in academia is not only a matter of justice, but can also be epistemically beneficial, and finally formulates four desiderata for change.

## 1 Introduction

The university has a problem with social class. Actually, it has two problems with class. The first one is that the university as an institution is still largely impervious to people from lower socio-economic backgrounds. In particular, people with middle and upper-middle-class backgrounds are overrepresented especially in very desirable tenured positions. This is well-known to about anyone who spends a few seconds reflecting on it. Fortunately, we also have hard research to back this up. Abramitzky et al. (2024)^1^ recently published the following numbers:

“We find a stark underrepresentation of individuals from lower socio-economic backgrounds: those born to parents in the bottom quintile of the parental income distribution account for <5% of all academics. […] Children born to the highest-earning fathers are particularly overrepresented, with those born to fathers in the 100th percentile having a 56% higher chance of becoming an academic than those born to fathers in the 99th percentile.”(see text footnote 1)

These numbers are simultaneously remarkable, yet not surprising to anyone who has spent a certain amount of time in the academic system. And not only that, even if people from lower socio-economic strata become academics, their papers still “receive fewer citations” (ibid.).

But curiously, the profession itself does not care about it. This is *weird*, to say the least. Consider for how long and how fervently academics have been drumming up support for women and non-white people to be included in the university. But when it comes to students and faculty with working-class backgrounds—we usually get radio silence. The practical results of this decades long struggle are, for example, highly-efficient affirmative action quotas. Regardless of whether one likes them or not: they are effective. But the result of selecting for gender and race is all too often that now not the lawyer's son, but the lawyer's daughter gets admitted to the Ivy League grad school. I am sure this is social progress, but it is not the level of social progress we should be content with. Regarding the silence on class, academia has received a one-two punch over the last decades. The first punch: the academic landscape has become increasingly depoliticized. This depoliticization appears on both the level of research and academics' labor practice. On the one hand, in the societal upheavals of the 1960s and 1970s, individual researchers were said to be more inclined to link their own research practice to certain reflections on social issues. On the other hand, academics as laborers seem to have been brought into line. The willingness to protest for better work conditions (for themselves or others) or to organize in unions has been on a steady decline since the 1990s throughout departments at university (at least in the West). The second punch lies with the according general shift away from class-consciousness to a focus on race and gender issues.

And this is the second, perhaps more profound problem: academia now simply ignores socio-economic class. This is ironic particularly because class differences have never been as absurdly extreme in the history of humankind as they are now. As of 2024, the top 8 people have as much wealth as the bottom 3,600,000,000 billion people. Each of these groups have 350 Million British pounds. University professors claiming to work on issues surrounding social justice are surprisingly ok with this. Sure, if you asked them directly, they would certainly be shocked at these numbers. But if you look at the topics they research and their publication habits, it turns out they actually do not mind that much. At least, it seems they deem combatting microaggressions more worthy their time and effort than considering economic inequality.

There used to be a time when Marxism is a current of thought was still so strong and influential that at least some academics in the humanities actively considered class as part of the ramifications of their own research. Of course, it is not the case that academics nowadays *never* talk about class issues since certain remnants of Marxist vocabulary have made their way into ordinary parlance (e.g., *Lumpenproletariat, Basis, und Überbau*). But it is still the case that reflection on class in the wake of Marx does not have the same standing it used to have just decades ago. This is contrary to talking points pertaining to “Cultural Marxism”. The recently much invoked Specter of “Cultural Marxism” is just a boogeyman. It is a boogeyman in the sense that most people accused of being “Cultural Marxists” have actually read very little of Marx's thought, if any. This has radically changed since around the late 80s to early 90s, at first in US academia, then gradually swapping over to the rest of the Western world. Class has become gradually overshadowed—meaning: effectively replaced—by race and gender as a critical category in our theorizing about the social world. Sure, intersectional academics may often enumerate class as part of the “umbrella of oppression”, but that is almost never more than lip-service. The reason we know it is lip-service is due to the fact that virtually no one is calling for effective real-world measures to combat injustice based on socio-economic class. This is particularly true in Western academic philosophy, but I am sure that many of these aspects can be generalized to the university as a whole.

## 2 Chronic class amnesia

How did we come to collectively ignore and forget about class? I think there are a few interlocking reasons. First, feminist standpoint epistemology has had an iron grip on socially progressive thought in the humanities. Standpoint epistemology is characterized by the following two theses. The situated knowledge thesis holds that social location—in terms of factors like gender, race, class, or other dimensions of identity—affects what we can know. The epistemic privilege thesis holds that unprivileged social positions are likely to generate perspectives are less biased than perspectives generated by other social position. Consider that stochastically large quantity of scholars in agenda defining positions in the humanities are at least middle-class. This group maybe marginalized in virtue of gender, race, or disability, but not socio-economic class. Taken together with the idea of standpoint epistemology (which many of those faculty subscribe to in one form or another), we can see that their class membership affords them a kind of class blindness in the same way that being white may afford one race blindness. Middle-to-upper middle-class faculty simply does not have to care about socio-economic issues in the same way that a white person does not really have to care about race.

This, secondly, demonstrates that the previously mentioned ignorance is willful. Willful ignorance is the deliberate avoidance or refusal to acquire knowledge that is relevant or morally significant in a way that would compel a person to act in ways they wish to avoid. Ignorance is commonly an active pursuit rather than a passive oversight. Ironically, we can apply these feminist grievances about willful ignorance to class. This ignorance about class is often actively upheld and excused through what I call classist figleaves. Philosophers have recently talked about racial and sexist figleaves. A racial figleaf is an excuse used to shield from accusations of racism while still allowing the underlying racist implications to persist, while sexist figleaves are the analog for sexism. For example, people may sometimes insist that “I don't hate women, I am married to one!” in an attempt to excuse misogynistic behavior. Accordingly then, a classist figleaf is an excuse used to shield from accusations of classism while still allowing the underlying classist implications to persist.

The most salient classist figleaf in academia lies in intersectionality. The idea of intersectionality insists that one's social markers “intersect” in order to create a unique pattern of oppression. This idea was partially introduced in an effort to combat what is sometimes perceived as Marxist class reductionism. The reality, unfortunately, is that intersectionality has allowed academics to consistently ignore class while maintaining that class is “included” or “thought together with” complaints about racial and gender-based discrimination. The pernicious classist figleaf then consists in speech acts that shut down focuses on socio-economic class by stating that “class is already included in intersectional approaches”, as it were. In reality, this usually simply drowns out questions regarding economic inequality.

## 3 Re-centering class

We can even come up with a few rough-and-ready reasons why social class ought to be centered both in our thought on the social world and in our efforts for social justice. The first reason it simply is the right thing to do. This might be disappointing to some, but it is difficult to further elaborate this point. If one at this point in time does not already accept that people from underserved socio-economic strata deserve better lives, there is no argument I can come up with that would be convincing to them. It would be similarly difficult to make someone understand why torturing puppies for fun is wrong.

A second reason is, again, due to standpoint epistemology. Almost exclusively focusing on gender and race (and sometimes disability), standpoint epistemologists insist that one's place in society at least partially and at least prima facie determines one's epistemic opportunities and capabilities. In other words, what you *are* (in some sense) pre-determines what you *know*. Marx certainly thought deeply about the epistemic ramifications of class, but again, however, the idea of standpoint epistemology is rarely extended to matters of social class nowadays. In what sense may, for example, philosophers from a working-class background produce different insights from others? Consider a possible parallel between comedians and philosophers. Perhaps the majority of the absolute best comedians of all time grew up poor and working class. Unfunny industry plants like Amy Schumer and Chris D'Elia, on the other hand, arguably owe the majority of their success to their background and the industry connections they were born into. Of course, there are exceptions. But the fact that people like Chris Rock, Dave Chappelle, or Nick Mullen are so funny is partly due to the strife they experienced growing up poor (of course there are exceptions too). Comedians who grew up in poverty see the world differently and often more accurately; often, the right kind of things, overlooked by others, appear salient to them. And they have been forced from a young age to engage with the world differently, usually through sardonic irony.

Now we should be quick to clarify that many academic philosophers, in my experience, have a somewhat underdeveloped sense of humor. But truly outstanding philosophy often also requires the same kind of broad worldview as standup comedy. People from lower socio-economic backgrounds at least sometimes have experiences specific to their backgrounds that may allow them to see the perennial problems of philosophy differently. Many philosophy professors of illustrious backgrounds never had to experience the struggles some others in the professions did and do. And this diversity of socio-economic backgrounds can itself prove to be an asset for us all. Luckily, we also have data supporting this intuition: “academics from lower socio-economic backgrounds are more likely to pursue research agendas off the beaten path” (ibid.).

## 4 What can be done?

It is not easy to pin down ways in which we could combat the class blindness currently rampant in academia. I am enumerating here a few steps for faculty to adopt.

1. Reconsider what you think Diversity is.

Center class as the focus of thought and action on social inequality, rather than just gender or race. Nobody wants to play different forms of discrimination against one another, but consider that the white male graduate student who lives in the unsafe part of town may—at least in some ways—in fact struggle more than a non-white graduate student who is fully supported by her affluent parents, living in a nice apartment close to campus her parents bought for her.^2^ But more importantly, make class relevant in job hiring, grad school application and all other areas where we are currently considering gender, race, and disability as relevant factors. We have no trouble considering race and gender as noteworthy aspects in question of equality. But when it comes to class, we as a profession are collectively blanking.

In other words, we need to reconsider the currently dominant ways of what diversity is. Many departments (at least in the West) will pride themselves in and advertise having an exceptionally high number of female and non-white students and staff. In many cases, highly ranked universities will simply attract middle-to-upper class individuals from different cultural and ethnic backgrounds to the effect that, in the end, children of rich parents rub shoulders with other children of rich parents, even if their skin colors and genders may be more diverse now than they were fifty years ago. But class is rarely, if ever a point of consideration in these matters. We rarely ask: can first-generation academics succeed at this institution? Are they looked out for? Are they given the same chances? This needs to change.

2. Support (or start) local initiatives.

Depending on where you live, there might be local initiatives you can join. For example, *ArbeiterKind.de* is a German non-profit organization dedicated to supporting students from non-academic backgrounds as they pursue higher education. Founded in 2008, its mission is to empower first-generation university students by providing guidance, resources, and mentorship to navigate the challenges of academia. Some colleges have dedicated initiatives for first-generation students. Consider getting involved. And if you do, help keep them *political* by raising class consciousness and resist institutional attempts at personalizing or individualizing the system problems we face.

3. Familiarize yourself with class differences in academia

Universities as highly complex institutions are often confusing to the uninitiated by design. In Germany in particular, universities are largely inscrutable entities which are difficult to navigate by design. Unlike universities in most Anglophone settings, German universities are largely tuition-free (which is nice), but students are left to their own devices in trying to understand their inner workings. In this sense, German universities are particularly bureaucratically hostile. Many people, in particular first-generation academics, fail or do not complete their degrees because they despair in steering the absurdities of German academia.

One of the main aspects that makes the university so difficult for first-generation academics is that *there is no one to ask*. And if you find someone to ask your question, they will promptly let you know that someone else is in charge, but they often cannot (or will not) tell you who that might be. Being on one's own in addition to the fact that many first-generation academics' parents are less than supportive of their child's choice to study, many students understandably faulter and leave the university, or they finish an undergraduate degree, but are too alienated to pursue another degree. Succeeding as a first-generation academic is then often more about grit and shrewdness than it is about academic merit or talent.

In other words, in order to understand how a university works, you often need someone to tell you how it works as an institution. For some lucky ones, this role falls to their parents who perhaps already graduated college a few decades past. But for students from working-class backgrounds, they are usually left on their own. This is not only for students. In the unlikely case that a student perseveres enough to earn a Ph.D. and obtain a position as faculty, the riddles do not cease, they increase, if anything. Arcane instructions from the dean, complicated and indirect department politics, performative meetings with unspoken codes of conduct all serve to further alienate the uninitiated.

4. Accept differences.

Academics usually have no (openly expressed) issues embracing cultural differences in their institutions; if anything, it is considered a sign of worldliness. Unfortunately, the same grace is in my experience not extended to first-generation philosophy students and colleagues. As Pierre Bourdieu taught us long ago, class differences are also essentially upheld through taste, in particular through the power to declare certain tastes as illegitimate. This works surprisingly open and unabashed in many philosophy departments. Theater, opera, classical music, auteur film, reading novels, classical dance, climbing, horse-riding, tennis, hiking, swimming are considered admirable ways to spend your free time. Video games, Hollywood cinema, heavy metal, TV, weightlifting are lowbrow interests you'd better keep to yourself. It is not rare even for the most self-aware for professors to scoff at such “lower” types of entertainment or forms of human life in general. This is likely because classism is one of the last de facto acceptable form of prejudice. Remind yourself that the way we consume things and spend our free time is often determined through copying what our parents did and what they afforded us, forming habits early that are often set for life. If you have respect for someone from a different culture eating food you might not like, extend the certain respect to those whose lifestyle, sport, and media consumption looks different from yours.

## 5 Barriers to change

There are, unfortunately, a number of barriers to changing the academic landscape for first-generation students and faculty. These barriers are both systematic and interpersonal. On the systematic level, I have argued in a previous article^3^ that the omission from class considerations in academic philosophy is a case of epistemic injustice. The most pernicious aspect of our collective omission is that we play into the hands of those who are most interested us not thinking about class at all. We are doing regressive ideologues and conservative think-tanks of this world a huge favor by collectively pretending class is not as big of a deal as it actually is. On an interpersonal level, many colleagues show resistance to acknowledging the real-world issue of class-based injustice. This resistance can take a number of different forms. The most effective form is likely simply not paying attention and carrying on as per usual.

Perhaps the most annoying (annoying to me, at least) form of resistance often comes in the form of “chin up!” comments or attempts at consolation. Bringing up the topic of class justice, some then say that I could be particularly proud of myself because I have achieved “so much”, as it were, especially against the odds. Such comments are well intentioned, but I cannot stand them. Compared to many others in my peer-group, I have had to spend more time, work up my nerves, swallow disappointments, and endure embarrassments than those from “academic households”. I don't see it as particularly admirable that my energy has been sapped unnecessarily this much over the years. There is nothing noble in poverty and precarity. Such supposed “nobility” is a smokescreen of the supposed meritocracy that academia pretends to be. Ultimately, such comments just serve to redirect attention from the systemic issues surrounding class in academic philosophy back onto conduct of individuals.

Many would gladly give up all this pride they are supposed to feel if in exchange they get to have an easier time existing in academia. Rather than trying to console those who suffer through them, we'd do better to put effort into changing these circumstances.

## Data Availability

The original contributions presented in the study are included in the article/supplementary material, further inquiries can be directed to the corresponding author.

## Footnotes

^1^ https://www.nber.org/system/files/working_papers/w33289/w33289.pdf

^2^This example is deliberately phrased this way to appear provocative in order to drive the point home about how much economic power can commonly override other aspects of social identity. Of course, it is a very real and tangible problem that in many countries, wealth inequality often accords to racial divisions. For example, in the USA this effect is most pronounced with African-Americans who due to historical and contemporary oppression lack the wealth that White people, but also other some other minority groups, have.

^3^ https://social-epistemology.com/wp-content/uploads/2022/09/spiegel_injustice_of_injustice_9-30-2022.pdf

